# Influence of Cardiac Motion on Stent Lumen Visibility in Photon-Counting CT Employing a Pulsatile Heart Model

**DOI:** 10.3390/diagnostics16121775

**Published:** 2026-06-09

**Authors:** Nils Petri, Henner Huflage, Julius F. Heidenreich, Jan-Peter Grunz, Christoph Panknin, Martin Petersilka, Thorsten A. Bley, Bernhard Petritsch

**Affiliations:** 1 Department of Internal Medicine I, University Hospital of Wuerzburg, Oberduerrbacherstr. 6, 97080 Wuerzburg, Germany; 2 Department of Diagnostic and Interventional Radiology, University Hospital of Wuerzburg, Oberduerrbacherstr. 6, 97080 Wuerzburg, Germany; huflage_h@ukw.de (H.H.); heidenreic_j@ukw.de (J.F.H.);; 3 Siemens Healthineers, Siemensstr. 3, 91301 Forchheim, Germany; 4 Department of Diagnostic and Interventional Radiology, Klinikum Klagenfurt am Woerthersee, Feschnigstraße 11, 9020 Klagenfurt am Wörthersee, Austria

**Keywords:** computed tomography, cardiac motion, photon-counting CT, coronary stents, stent lumen visibility

## Abstract

**Introduction:** Detection of in-stent restenosis by cardiac CT is challenging due to blooming artifacts. The technological progress of CT scanners and especially the recent introduction of photon-counting detectors (PCDs) has led to an improvement in image quality. Several studies have analyzed the lumen visibility of coronary stents, but most studies used models which did not simulate cardiac movement. In this study we use a pulsatile heart model to simulate a heartbeat to analyze the effects of cardiac motion on image quality. **Methods:** Seventeen different coronary stents with an outer diameter of 3.0 mm were implanted into polyolefin tubes. The tubes were then filled with diluted contrast medium and attached to the pulsatile heart model. The stents were scanned in a third-generation dual-source CT with an energy-integrating detector (EID) and a first-generation PCD CT. **Results:** In motion, the mean visible stent lumen was reduced from 64.4% to 59.4% in EID CT, from 61.4% to 56.0% in PCD CT using the Bv60 kernel, and from 72.9% to 62.9% in PCD CT using the Bv72 kernel, each in standard resolution mode. Employing the ultra-high-resolution mode (UHR), stent lumen visibility was reduced from 61.3% to 57.9% with the Bv60 kernel and from 71.7% to 61.8% with the Bv72 kernel. The difference between static imaging and motion was significant in each instance (*p* < 0.001). **Conclusions:** While PCD CT and the use of sharper kernels improves the image quality in comparison with EID CT and smoother kernels, the impact of cardiac motion on the reduction in stent lumen visibility is substantial. Hence, the best image quality is achieved in patients with a normal and regular heart rate. If this is not possible to achieve, a retrospective acquisition mode should be considered.

## 1. Introduction

Computed tomography coronary angiography (CTCA) is a class I recommendation for the detection of coronary artery disease (CAD) in symptomatic patients with a low or moderate (>5–50%) pretest probability [1]. However, accurate quantification of lesions in patients with known CAD remains challenging as image quality is often impaired by blooming artifacts, beam hardening and partial volume effects in the presence of calcified lesions or coronary stents [2,3,4,5].

The recent introduction of photon-counting detector (PCD) CTs offer improved spatial resolution, reduced electronic noise, increased contrast-to-noise ratio, and the availability of spectral information in comparison to energy-integrated detector (EID) systems [6].

These improvements in the PCD are achieved by a new detector, which employs a semiconductor [7].

Several phantom and clinical studies have demonstrated the benefits of photon-counting CT for coronary plaque and stent imaging [8,9,10,11,12,13]. Ultra-high-resolution PCD-CT has been shown to improve coronary stenosis quantification and reduce blooming artifacts from calcified plaques compared with conventional EID systems [9,12,13]. These phantom studies reported improved visualization of coronary stent lumina and reduced stent-related blooming artifacts owing to the higher intrinsic spatial resolution of photon-counting detectors. Early patient studies have confirmed the feasibility of coronary stent assessment using ultra-high-resolution PCD-CT and demonstrated improved delineation of small coronary structures and plaque components [8,10,11].

However, most available studies have focused on static phantom setups or on image quality metrics acquired under relatively controlled conditions. Studies investigating whether the technical advantages of photon-counting CT are maintained in the presence of cardiac motion remain limited. This question is particularly relevant for coronary stent imaging, where motion artifacts and blooming artifacts may interact and jointly determine lumen visibility.

Cardiac motion, particularly in patients with an elevated or irregular heart rates, can negatively affect image quality [14].

While new detector technologies significantly increase spatial resolution, the nominal temporal resolution of dual-source PCD systems remains similar, compared to dual-source EID systems with 66 ms per frame. However, robust data about the benefits of new detector technologies during the motion phase of cardiac cycle remain sparse. The purpose of this study was the systematic analysis of the influence of cardiac motion comparing modern EID and PCD CT systems, free from additional in vivo confounders such as involuntary breathing motion [7,15,16]. In the PCD CT, the ultra-high-resolution (UHR) mode does not rely on post-patient collimation by a comb filter as in the EID-CT, but instead uses smaller detector pixels, which enables a higher spatial resolution without loss of dose efficiency. At identical dose levels, this mode has also been shown to provide an improved signal-to-noise ratio compared to standard resolution, as demonstrated by numerous studies [7,15,16]. The PCD can improve spatial resolution and edge definition even in standard-resolution acquisition. In addition, the availability of a dedicated ultra-high-resolution mode provides a further resolution reserve that is particularly relevant for small, high-contrast structures such as coronary stents. Against this background, our phantom setup allows us to disentangle motion-related blurring from detector- and reconstruction-driven gains in detail visibility across EID, PCD in standard resolution, and PCD in UHR.

To this end, we employed a pulsatile heart model to assess the influence of cardiac motion on stent lumen visibility in a dual-source PCD CT system compared with a third generation dual-source EID CT, both providing identical temporal resolution.

## 2. Methods

### 2.1. Experimental Setup

**Figure 1:**
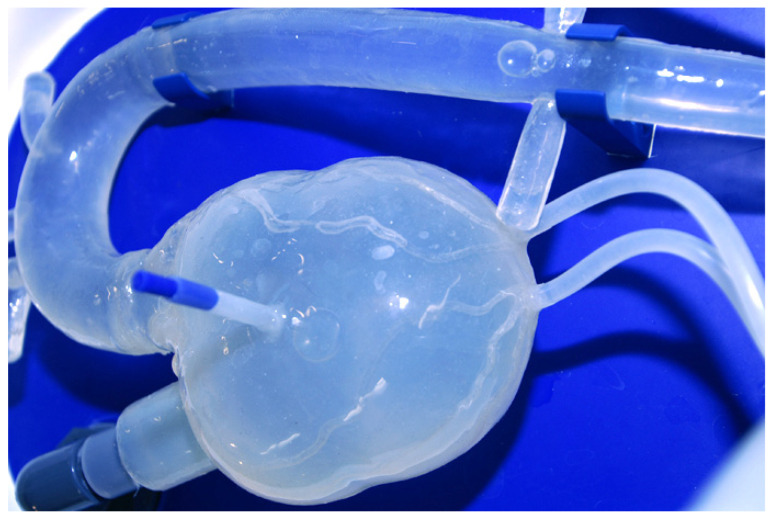
Close up of the phantom heart model employed in this study (CoroSim^®^; Mecora, Aachen, Germany). This anatomical heart model is made from silicone and is filled with fluid. A pump creates a pulsatile flow, which in turn causes the alternating expansion and contraction of the model, thus simulating the beating of a heart. The tubes containing the coronary stents were attached to the outside of this model.

We included 17 different stents, each with an outer diameter of 3.0 mm, in this study. The stents varied in material, structure, coating, and strut thickness. The stent characteristics are listed in Table 1. The stents were implanted into polyolefin tubes at the pressure specified by the manufacturer to achieve an outer diameter of 3.00 mm. The tubes were then filled with contrast medium diluted to a density of 350 HU at 120 kV to mimic the density of contrast enhanced blood (Imeron 300; Bracco Imaging, Konstanz, Germany) and then attached to the pulsatile heart model (CoroSim; Mecora, Aachen, Germany) in a position resembling the course of the left anterior descending coronary artery. This silicone heart model is connected to a pump that fills the heart chamber with water, and by setting the pump to pulsatile mode, it mimics the expansion and contraction of a heart (Figure 1). The model has a fixed heart rate of 75/min, so we used an electrocardiogram (ECG) simulator set to a sinus rhythm with a frequency of 75/min, which was connected to the CT for ECG triggering.

### 2.2. CT Scan Protocol

The study was performed on a third-generation dual-source EID CT (SOMATOM Force; Siemens Healthineers, Forchheim, Germany) and a first-generation PCD CT (NAEOTOM Alpha; Siemens Healthineers, Forchheim, Germany). The stents were aligned parallel to the scanner *z*-axis and scanned using retrospectively ECG-gated cardiac spiral acquisitions at a fixed tube potential of 120 kVp. The gantry rotation time was 0.25 s with a pitch of 0.25, which represents the shortest available rotation on both systems and results in an effective temporal resolution of 66 ms for cardiac imaging.

### 2.3. Image Reconstruction

For both detector types, raw data were reconstructed with identical in plane geometry (512 × 512 matrix, 50 mm field of view). On the EID CT, the effective tube current–time product was 200 mAs (CTDI_vol_ = 42.7 mGy), with a slice thickness of 0.6 mm and an increment of 0.3 mm. Images were reconstructed with the Bv59 kernel and strength level 3 of ADMIRE (Siemens Healthineers), which represents the sharpest vascular kernel on the SOMATOM Force and provides a spatial frequency of approximately 8.3 lp/cm at 50% modulation and 11.7 lp/cm at 10% modulation.

For the PCD CT in SR mode, the effective tube current–time product was 140 mAs (CTDI_vol_ = 41.8 mGy), with a slice thickness of 0.4 mm and an increment of 0.2 mm. In UHR mode, the tube current–time product was likewise 140 mAs (CTDI_vol_ = 41.7 mGy), while the slice thickness was reduced to 0.2 mm with an increment of 0.1 mm. Despite the different nominal mAs settings between EID and PCD, comparable CTDI_vol_ values were achieved because the effective mAs is determined by tube current, rotation time, and pitch, and the pitch itself depends on the respective collimation of each system.

For PCD CT, the Bv60 kernel was selected to approximate the clinical sharpness of the Bv59 vascular kernel on the EID CT in SR mode. Furthermore, an additional sharper kernel (Bv72) was evaluated in PCD UHR acquisitions. The PCD with the Bv60 and the Bv72 kernel provides spatial frequency of approximately 8.8 lp/cm and 14.1 lp/cm at 50% modulation and 11.9 lp/cm and 14.1 lp/cm at 10% modulation, respectively. While the UHR mode allows for substantially higher number of voxels and a higher nominal spatial resolution, reducing the slice thickness from 0.4 mm to 0.2 mm at similar CTDI_vol_ markedly increases image noise. When identical reconstruction kernels and parameters (including slice thickness) are applied in SR and UHR, this “small pixel effect” can dominate, such that the theoretical gain in resolution is partly offset or even outweighed by the noise increase, particularly in strictly axial stent configurations, where the smallest voxel size may not result in a meaningful diagnostic advantage [16,17]. Moreover, the effect of iterative reconstruction should not be regarded as a linear or isolated modifier of image noise. Rather, its denoising behavior may depend on the underlying acquisition and reconstruction setting, including detector resolution mode, kernel sharpness, and voxel geometry; therefore, a nominally identical iterative reconstruction level may not exert an identical effect in SR and UHR reconstructions.

The present study was designed to compare clinically optimized acquisition and reconstruction settings for both detector technologies rather than strictly parameter-matched reconstructions. Consequently, the observed differences in stent lumen visibility reflect the combined influence of detector design, reconstruction kernel characteristics, and slice thickness.

### 2.4. Image Analysis

Syngo.via (Siemens Healthineers, Forchheim, Germany) was used for image analysis. The manual measurements were made in the axial reformations with the window width set to 1500 HU and the center to 300 HU. The longitudinal reformations were supplied for demonstration purposes only. Measurements were taken at maximum expansion of the heart, resembling the end diastolic phase. At this time, the stent is not moving and therefore represents the static phase. Maximum motion was defined as the mid-point between full expansion and full contraction. Measurement of the visible lumen diameter, the attenuation, and noise were made by one reader (N. P.) in three axial slices with 13 years of experience in cardiovascular CT imaging.

### 2.5. Statistical Analysis

Statistical analyses were performed using dedicated software (SPSS Statistics, version 28.0; IBM, Armonk, NY, USA). The paired Student’s *t*-test was used to analyze statistical significance, which was assumed at *p* < 0.05. We reported the two-sided *p*-values. Measurement results are presented as mean, standard deviation and range.

## 3. Results

### 3.1. Influence of Motion on Visible Stent Lumen Diameter

The mean visible lumen diameter of the stents at rest in the EID CT using the Bv59 kernel was 64.4% ± 8.0%. Under motion, the visible lumen diameter was merely 59.4% ± 9.1% (*p* < 0.001). In the PCD CT using the Bv60 kernel in SR mode, the mean visible lumen diameter of the stents at rest was 61.4% ± 8.6% and 56.0% ± 7.2% in motion (*p* < 0.001). Switching from SR to the UHR mode with the same kernel produced a mean visible lumen diameter of 61.3% ± 6.8% at rest and 57.9% ± 5.1% in motion (*p* < 0.001). Using a sharper kernel (Bv72), the stent lumen visibility at rest was 72.9% ± 7.1% (SR mode) and 71.7% ± 6.1% (UHR mode). In motion, the lumen visibility was reduced to 62.9% ± 8.3% and 61.8% ± 8.9%, respectively. In both cases, the difference between motion and rest was significant (*p* < 0.001). The results are shown in Table 2.

### 3.2. Comparison of Standard and Ultrahigh Resolution Modes and the Different Kernels

In the PCD CT, the difference in stent lumen visibility between SR and UHR mode with the Bv60 kernel at rest (*p* = 0.900) and in motion (*p* = 0.160) was not significant. There was also no significant difference between SR and UHR mode for the Bv72 kernel (at rest: *p* = 0.180, in motion *p* = 0.400). Comparing the Bv59 kernel in EID CT with the Bv60 kernel in PCD CT in SR mode, no significant differences were found for the lumen visibility of the stents at rest (*p* = 0.100), but a statistical difference was observed in motion (*p* = 0.049). A statistical difference was also found for the comparison of the Bv59 kernel in EID CT and the Bv60 kernel in PCD CT when scanning in UHR mode at rest (*p* = 0.005). However, there was no statistically significant difference in the lumen visibility in motion (*p* = 0.250). Comparing the Bv59 kernel in EID CT with the Bv72 kernel in PCD CT, we found a significant difference in lumen visibility at rest (both SR and UHR mode: *p* < 0.001). But comparing these kernels in motion, we only found a significant difference in SR mode (*p* = 0.009), but not in UHR mode (*p* = 0.193). Additionally, significant differences were ascertained between Bv60 and Bv72 in both scan modes at rest (*p* < 0.001) and between BV60 and BV72 in motion (SR: *p* < 0.001, UHR: *p* = 0.015).

### 3.3. Comparison of Stents with Different Strut Thicknesses

To determine the influence of strut thickness, we divided the stents into two groups, stents with a strut thickness of over 0.08 mm and stents with a strut thickness of under 0.08 mm. We did not find a significant difference between the two groups for Bv59 and Bv60 in SR and in UHR mode, Bv72 in SR at rest and in motion, and Bv72 UHR at rest. We only found a significant difference with the lumen visibility of differing stent struck thicknesses in UHR mode in motion. The stents with a thinner strut thickness showed a higher lumen visibility.

## 4. Discussion

**Figure 2:**
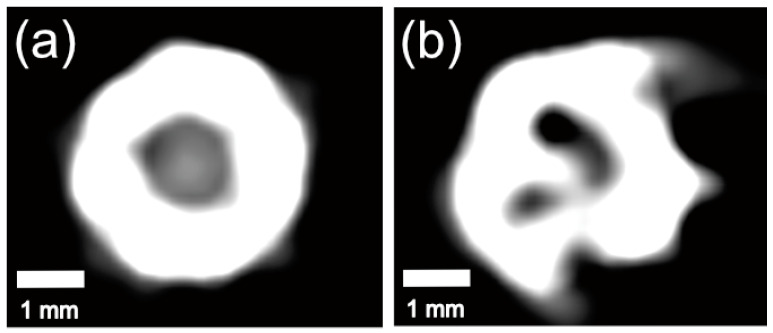
Axial slice of a stent (Boston Scientific Promus Element (Boston Scientific Corporation, Marlborough, MA, USA)) at rest (**a**) and in motion (**b**). PCD CT in UHR mode using the Bv60 kernel. The window width was set to 1500 HU and the center to 300 HU.

**Figure 3:**
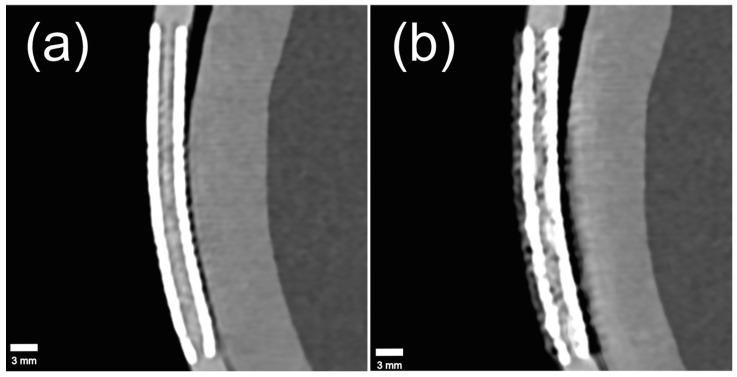
Longitudinal slice of a stent (Boston Scientific Promus Element (Boston Scientific Corporation, Marlborough, MA, USA)) at rest (**a**) and in motion (**b**). PCD CT in SR mode using the Bv60 kernel. The window width was set to 1500 HU and the center to 300 HU. The longitudinal slice is just for demonstration purposes to better show the motion artifacts. The measurements were made in the axial slices.

**Figure 4:**
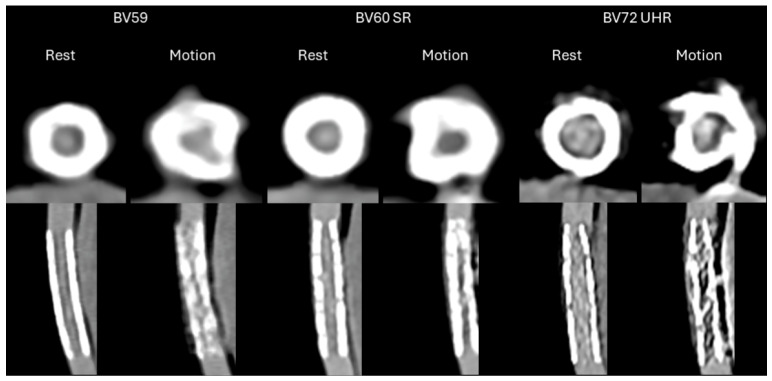
Longitudinal and axial slices of a stent (Braun Coroflex Blue (B. Braun SE, Melsungen, Germany)) at rest and in motion scanned with the EID CT, reconstructed with a Bv59 kernel, and scanned with a PCD CT in SR mode using the Bv60 kernel and using the Bv72 kernel in UHR mode. The window width was set to 1500 HU and the center to 300 HU.

In this study, a pulsatile heart model was used to simulate cardiac motion and evaluate its impact on stent lumen visibility in both photon-counting detector (PCD) CT and latest generation energy-integrating detector (EID) CT. We observed a significant reduction in stent lumen visibility caused by cardiac motion across all reconstruction kernels and in both EID and PCD CT, including standard resolution (SR) and ultra-high resolution (UHR) modes. In addition to the reduced stent lumen visibility, the stent images acquired under motion also demonstrated motion-related artifacts, as illustrated in Figure 2, Figure 3 and Figure 4. The reduction in stent lumen visibility by cardiac motion was similar between both CT scanners, which aligns with the fact that PCD CT does not have an improved temporal resolution compared to EID CT as the gantry rotation time—one of the main factors for temporal resolution—is limited to 0.25 s for both scanners, yielding 66 ms temporal resolution due to the dual-source technology.

The EID CT employed the Bv59 kernel, the sharpest vascular kernel available on the SOMATOM Force. The PCD CT allows the use of sharper kernels without a substantial increase in noise; using such kernels, the PCD CT demonstrated slightly improved stent lumen visibility at rest. However, this benefit is relatively small and is outweighed by the detrimental impact of cardiac motion. Notably, we did not observe a statistically significant difference in lumen visibility between UHR and SR modes when using the same reconstruction kernel. Although thinner reconstructions (0.2 mm vs 0.4 mm) would theoretically be expected to increase image noise because fewer photons contribute to each reconstructed slice, this effect was not observed in our UHR datasets.

This phenomenon highlights the complexity of the so-called “small pixel effect”—whereby reducing voxel size without increasing dose may theoretically increase image noise and thereby offset potential gains in spatial resolution, particularly for stents aligned strictly axially. However, in the present study, image noise in UHR reconstructions did not increase despite the thinner slice thickness, suggesting that additional reconstruction-related factors substantially influenced the final image appearance [17,18].

The observed differences in visible stent lumen diameter and image noise should therefore be interpreted in the context of a complex reconstruction process involving several interdependent parameters. Final image noise is influenced not only by slice thickness, but also by spatial resolution mode, reconstruction kernel, and quantum iterative reconstruction (QIR). Importantly, a nominally identical QIR level may not exert identical effects across standard resolution and UHR datasets; comparatively stronger noise suppression in UHR reconstructions may partly compensate for the theoretically expected noise increase associated with thinner slices. Consequently, the individual effects of slice thickness, kernel sharpness, spatial resolution mode, and QIR strength cannot be fully separated when interpreting image noise and visible stent lumen diameter.

This interpretation is supported by recent photon-counting CT studies demonstrating relevant interactions between QIR strength and reconstruction parameters. Krompaß et al. showed in photon-counting CTA that the effect of QIR on image noise, contrast-to-noise ratio, and image blurring depended on the reconstruction kernel, with stronger relative noise reduction observed for sharper vascular kernels [19]. Similarly, Vattay et al. systematically varied slice thickness, vascular reconstruction kernel, and QIR level in standard-resolution and ultra-high-resolution PCD-CCTA datasets and reported different optimal reconstruction settings for SR and UHR acquisitions [20]. These findings suggest that reconstruction parameters should be tailored to the specific diagnostic task rather than selected solely to maximize spatial resolution or minimize slice thickness.

Importantly, visible lumen diameter represents only one component of diagnostic performance in coronary stent imaging. In clinical practice, elevated image noise may reduce diagnostic confidence for the detection of subtle in-stent restenosis by obscuring low-contrast intraluminal abnormalities or mimicking fine soft tissue components. Conversely, stronger iterative noise suppression may improve quantitative noise metrics while potentially altering image texture and fine structural detail. Therefore, the reconstruction setting that maximizes visible stent lumen diameter may not necessarily correspond to the setting that optimizes diagnostic accuracy [21]. Achieving the optimal balance between spatial resolution, image noise, and iterative noise suppression remains an important consideration for clinical protocol optimization.

Although the strut thickness of the stents is considered an important determinant of blooming artifacts, we observed only limited differences between thin- and thick-strut stents. This may reflect the relatively small number of stents per subgroup and the heterogeneity of other design features, including stent geometry, alloy composition, and coating, which may influence lumen visibility independently of strut thickness.

These findings emphasize that cardiac motion remains a primary determinant of image quality in coronary stent imaging, even in the era of photon-counting detector technology and advanced reconstruction. In this context, the optional UHR acquisition mode represents an important and timely development for coronary stent imaging, as smaller detector pixels and dedicated high-resolution reconstruction can improve delineation of fine metallic structures [22,23]. However, UHR does not mitigate motion-related artifacts per se; rather, it increases the need for motion-minimized acquisition conditions because the higher spatial frequency content and thinner effective slice reconstructions render residual motion more conspicuous.

Moreover, UHR entails relevant acquisition trade-offs in current dual-source PCD-CT implementations. Compared with standard collimation (144 × 0.4 mm = 57.6 mm z-coverage), UHR is typically associated with a substantially reduced z-coverage (120 × 0.2 mm = 24 mm). Particularly for ECG-gated protocols, the smaller collimation can translate into longer acquisition times and a higher number of acquisition blocks to cover the heart volume (e.g., requiring five instead of three blocks), potentially increasing susceptibility to additional motion and prolonging breath-hold demands and contrast bolus timing. While PCD systems may offer improved dose efficiency and can preserve signal-to-noise characteristics when reconstruction settings are appropriately adapted, the reduced z-coverage in UHR reflects current technical constraints, most plausibly driven by the markedly increased data density and readout/transfer requirements at the detector level. Consequently, although UHR is conceptually attractive and may represent a future standard for dedicated coronary stent assessment, its routine use in all cardiac CT acquisitions remains limited at present.

Beyond hardware-related improvements in spatial resolution, advanced motion correction algorithms may represent an additional strategy to reduce motion-related image degradation in future cardiac CT applications. Recent developments in subpixel and online motion correction techniques in other high-resolution vascular imaging modalities have demonstrated promising results and may provide conceptual frameworks for future cardiac CT motion compensation approaches [14,24,25].

This study has several limitations. First, this study was designed as a phantom experiment, and its transferability to a clinical setting is limited. Second, while the aim of this study was to isolate the effect of cardiac motion on image quality with the employed pulsatile heart model, in vivo exams might be confounded by additional respiratory motion, which was not investigated in this study. Third, the polyolefin tubes containing the stents and contrast medium were attached externally to the heart model and were not surrounded by any material mimicking pericardial fat and thoracic soft tissue. Fourth, the potential influence of the iterative reconstruction algorithm was not evaluated. Fifth, UHR acquisition and its specific trade-offs (e.g., reduced z-coverage) were not systematically assessed in the current setup. Sixth, the pulsatile heart model employed a fixed heart rate of 75/min and therefore does not fully represent the broad spectrum of physiological heart rates and rhythm irregularities encountered in clinical practice. Particularly in patients with tachycardia or arrhythmias, motion artifacts are expected to become more pronounced, potentially further reducing stent lumen visibility. Future studies including variable heart rates and irregular motion profiles are warranted to better characterize the performance of photon-counting CT under more challenging dynamic conditions. Finally, all measurements were performed by a single reader.

## 5. Conclusions

This study evaluated the isolated influence of cardiac motion on stent lumen visibility using a pulsatile heart model. While PCD CT and the use of sharper reconstruction kernels improve image quality compared with EID CT and less sharp kernels during static heart phases, the motion sensitivity and its effect on stent lumen visibility caused by cardiac contraction remains substantial. Thus, despite increased spatial resolution, optimal image quality still depends on acquisition during motion-free cardiac phases.

## Figures and Tables

**Table 1:** Characteristics of the coronary stents.

Number	Company	Name	Material	Coating	Strut Thickness, mm
1	Abbott (Vascular, Santa Clara, CA, USA)	Xience Pro	Cobalt–chromium alloy	Everolimus	0.081
2	Abbott (Vascular, Santa Clara, CA, USA)	Xience Xpedition	Cobalt–chromium alloy	Everolimus	0.081
3	Biosensors (International Group, Singapore, Singapore)	BioFreedom	Stainless steel	Biolimus	0.112
4	Biotronik (Berlin, Germany)	PRO-Kinetic Energy	Cobalt–chromium alloy	None	0.060
5	Biotronik (Berlin, Germany)	Papyrus	Cobalt–chromium alloy	None	0.060
6	Biotronik (Berlin, Germany)	Synsiro	Cobalt–chromium alloy	Sirolimus	0.060
7	Boston Scientific (Corporation, Marlborough, MA, USA)	Promus Element	Platinum–chromium alloy	Everolimus	0.081
8	Boston Scientific (Corporation, Marlborough, MA, USA)	Omega	Platinum–chromium alloy	None	0.081
9	Braun (SE, Melsungen, Germany)	Coroflex Blue	Cobalt–chromium alloy	None	0.065
10	Braun (SE, Melsungen, Germany)	Coroflex ISAR Neo	Cobalt–chromium alloy	Sirolimus	0.055
11	Cordis (Miami Lakes, FL, USA)	Cypher Select	Stainless steel	Sirolimus	0.140
12	Medtronic (Minneapolis, MN, USA)	Resolute Integrity	Cobalt–chromium alloy	Zotarolimus	0.091
13	Medtronic (Minneapolis, MN, USA)	Resolute Onyx	Cobalt–nickel alloy	Zotarolimus	0.081
14	Sorin Group (Milan, Italy)	Janus Flex	Stainless steel	Tacrolimus	0.065
15	Terumo (Corporation, Tokyo, Japan)	Ultimaster	Cobalt–chromium alloy	Sirolimus	0.080
16	Translumina (GmbH, Hechingen, Germany)	Yukon CC	Cobalt–chromium alloy	None	0.068
17	Biotronik (Berlin, Germany)	Magmaris	Magnesium alloy	BIOlute	0.150

**Table 2:** Mean visible lumen diameters in percentage in the static and motion phases, in-stent attenuation and noise. Values are provided as mean, standard deviation and range.

CT, Kernel and Resolution Mode	Visible Lumen Diameter Static	Visible Lumen Diameter Motion	Attenuation (HU)	Noise (HU)	Visible Lumen Diameter Static vs. Motion (*p*-Value)
EID Bv59	64.4% ± 8.0 (55.6–90.0)	59.4% ± 9.1 (47.8–86.7)	380.6 ± 48.6 (285.3–482.7)	18.7 ± 1.7 (16.0–22.0)	<0.001
PCD Bv60 SR	61.4% ± 8.6 (53.3–86.7)	56.0% ± 7.2 (40.0–75.6)	482.1 ± 36.4 (399.3–538.3)	15.5 ± 1.5 (13.0–18.0)	<0.001
PCD Bv60 UHR	61.3% ± 6.7 (53.3–83.3)	57.9% ± 5.1 (52.2–90.0)	424.2 ± 35.5 (345.0–483.0)	15.9 ± 1.6 (12.0–18.0)	<0.001
PCD Bv72 SR	72.9% ± 7.1 (64.4–92.2)	62.9% ± 8.3 (52.2–90.0)	518.4 ± 57.6 (418.3–623.3)	28.8 ± 4.1 (21.0–37.0)	<0.001
PCD Bv72 UHR	71.7% ± 6.1 (64.4–91.1)	61.8% ± 8.9 (50.0–90.0)	497.5 ± 92.1 (299.7–607.0)	22.3 ± 1.7 (17.0–26.0)	<0.001

## Data Availability

The raw data supporting the conclusions of this article will be made available by the authors on request.

## Abbreviations

CAD	Coronary artery disease
CTCA	Computed tomography coronary angiography
CTDIvol	Computed tomography dose index volume
CNR	Contrast-to-noise ratio
ECG	Electrocardiogram
EID	Energy-integrating detector
UHR	Ultra-high resolution
PCD	Photon-counting detector
SR	Standard resolution

## References

[1] Vrints C., Andreotti F., Koskinas K.C., Rossello X., Adamo M., Ainslie J., Banning A.P., Budaj A., Buechel R.R., Chiariello G.A. (2024). 2024 ESC Guidelines for the management of chronic coronary syndromes. Eur. Heart J..

[2] Dai T., Wang J.-R., Hu P.-F. (2018). Diagnostic performance of computed tomography angiography in the detection of coronary artery in-stent restenosis: Evidence from an updated meta-analysis. Eur. Radiol..

[3] Gassenmaier T., Petri N., Allmendinger T., Flohr T., Maintz D., Voelker W., Bley T.A. (2014). Next generation coronary CT angiography: In vitro evaluation of 27 coronary stents. Eur. Radiol..

[4] André F., Müller D., Korosoglou G., Hosch W., Kauczor H.-U., Katus H.A., Steen H. (2014). In-vitro assessment of coronary artery stents in 256-multislice computed tomography angiography. BMC Res. Notes.

[5] Eckert J., Renczes-Janetzko P., Schmidt M., Magedanz A., Voigtländer T., Schmermund A. (2019). Coronary CT angiography (CCTA) using third-generation dual-source CT for ruling out in-stent restenosis. Clin. Res. Cardiol..

[6] Flohr T., Petersilka M., Henning A., Ulzheimer S., Ferda J., Schmidt B. (2020). Photon-counting CT review. Phys. Med..

[7] Fix Martinez M., Klein L., Maier J., Rotkopf L.T., Schlemmer H.-P., Schönberg S.O., Kachelrieß M., Sawall S. (2023). Potential radiation dose reduction in clinical photon-counting CT by the small pixel effect: Ultra-high resolution (UHR) acquisitions reconstructed to standard resolution. Eur. Radiol..

[8] Geering L., Sartoretti T., Mergen V., Cundari G., Rusek S., Civaia F., Rossi P., Wildberger J.E., Templin C., Manka R. (2023). First in-vivo coronary stent imaging with clinical ultra high resolution photon-counting CT. J. Cardiovasc. Comput. Tomogr..

[9] Stein T., Taron J., Verloh N., Doppler M., Rau A., Hagar M.T., Faby S., Baltas D., Westermann D., Ayx I. (2023). Photon-counting computed tomography of coronary and peripheral artery stents: A phantom study. Sci. Rep..

[10] Kiani I., Mohebbi A., Jannatdoust P., Darmiani K., Mohammadzdeh S., Mohammadi A., Gholamrezanezhead A. (2025). Comparison of photon-counting CT angiography with energy-integrating CT angiography in coronary artery stenosis: A systematic review and meta-analysis. BMC Med. Imaging.

[11] Eberhard M., Candreva A., Rajagopal R., Mergen V., Sartoretti T., Stähli B.E., Templin C., Manka R., Alkadhi H. (2024). Coronary Stenosis Quantification with Ultra-High-Resolution Photon-Counting Detector CT Angiography. JACC Cardiovasc. Imaging.

[12] Petritsch B., Petri N., Weng A.M., Petersilka M., Allmendinger T., Bley T.A., Gassenmaier T. (2021). Photon-Counting Computed Tomography for Coronary Stent Imaging. Investig. Radiol..

[13] Michael A.E., Schoenbeck D., Becker-Assmann J., Niehoff J.H., Flohr T., Schmidt B., Panknin C., Baer-Beck M., Hickethier T., Maintz D. (2023). Coronary Stent Imaging in Photon Counting Computed Tomography: Optimization of Reconstruction Kernels in a Phantom. Eur. J. Radiol..

[14] Zhang L., Jiang B., Chen Q., Wang L., Zhao K., Zhang Y., Vliegenthart R., Xie X. (2022). Motion artifact removal in coronary CT angiography based on generative adversarial networks. Eur. Radiol..

[15] Huflage H., Hendel R., Woznicki P., Conrads N., Feldle P., Patzer T.S., Ergün S., Bley T.A., Kunz A.S., Grunz J.-P. (2024). The Small Pixel Effect in Ultra-High-Resolution Photon-Counting CT of the Lumbar Spine. Investig. Radiol..

[16] Huflage H., Hendel R., Kunz A.S., Ergün S., Afat S., Petri N., Hartung V., Gruschwitz P., Bley T.A., Grunz J.-P. (2023). Investigating the Small Pixel Effect in Ultra-High Resolution Photon-Counting CT of the Lung. Investig. Radiol..

[17] Graafen D., Halfmann M.C., Emrich T., Yang Y., Kreuter M., Düber C., Kloeckner R., Müller L., Jorg T. (2023). Optimization of the Reconstruction Settings for Low-Dose Ultra-High-Resolution Photon-Counting Detector CT of the Lungs. Diagnostics.

[18] Milos R.-I., Röhrich S., Prayer F., Strassl A., Beer L., Heidinger B.H., Weber M., Watzenboeck M.L., Kifjak D., Tamandl D. (2023). Ultrahigh-Resolution Photon-Counting Detector CT of the Lungs: Association of Reconstruction Kernel and Slice Thickness with Image Quality. Am. J. Roentgenol..

[19] Krompaß K., Goldbrunner F.A., Hartung V., Ergün S., Peter D., Hendel R., Huflage H., Patzer T.S., Hennes J.-L., Bley T.A. (2024). Combined influence of quantum iterative reconstruction level and kernel sharpness on image quality in photon counting CT angiography of the upper leg. Sci. Rep..

[20] Vattay B., Boussoussou M., Vecsey-Nagy M., Kolossváry M., Juhász D., Kerkovits N., Balogh H., Nagy N., Vértes M., Kiss M. (2024). Qualitative and quantitative image quality of coronary CT angiography using photon-counting computed tomography: Standard and Ultra-high resolution protocols. Eur. J. Radiol..

[21] Mergen V., Sartoretti T., Baer-Beck M., Schmidt B., Petersilka M., Wildberger J.E., Euler A., Eberhard M., Alkadhi H. (2022). Ultra-High-Resolution Coronary CT Angiography with Photon-Counting Detector CT: Feasibility and Image Characterization. Investig. Radiol..

[22] Hagar M.T., Emrich T., Vecsey-Nagy M., Bamberg F., Schlett C.L., Garrison G.W., Wenderoth A., Isaak A., Kuetting D., Luetkens J.A. (2025). Spectral ultrahigh-resolution photon-counting CT for coronary stent imaging: Evaluation in a dynamic phantom. Eur. Radiol. Exp..

[23] Occhipinti M., Clemente A., De Gori C., Positano V., Neglia D., Celi S., Meloni A., Berti S., Cademartiri F. (2026). Ultra-high spatial resolution at photon-counting computed tomography: Technical insights and sustainable applications in cardiothoracic imaging. Eur. Radiol. Exp..

[24] Zhang X., Zhong H., Wang S., He B., Cao L., Li M., Jiang M., Li Q. (2024). Subpixel motion artifacts correction and motion estimation for 3D-OCT. J. Biophotonics.

[25] Ma H., Wang Z., Zuo C., Huang Q. (2022). Three dimensional confocal photoacoustic dermoscopy with an autofocusing sono-opto probe. J. Biophotonics.

